# Supplementary material for: Mental Health and Health-Related Quality of Life in German Adolescents after the Third Wave of the COVID-19 Pandemic
Source: Children (Basel). 2022 May 25;9(6):780. doi: 10.3390/children9060780 (PMC9221692; doi:10.3390/children9060780)
Supplement: Supplementary file 1 [file children-09-00780-s001.zip › children-1734796-supplementary.pdf]

Table S1. Self-reported HRQoL and psychological symptoms in different sex groups.

	Girls	Boys	Diverse
	n=695	*n=674*	*n=13*
HRQoL[a]			
General HRQoL index, mean T values (SD)	51.5 (10.70)	56.2 (11.22)	42.2 (7.85)
SDQ[b], mean scores (SD)			
Emotional problems	3.8 (2.63)	2.2 (1.96)	6.2 (2.56)
Conduct problems	1.8 (1.56)	1.6 (1.50)	2.5 (1.57)
Hyperactivity	3.4 (2.22)	3.4 (2.21)	4.0 (2.45)
Peer problems	2.5 (1.85)	2.2 (1.80)	2.7 (1.56)
Prosocial behavior	8.4 (1.79)	8.1 (1.78)	7.0 (2.17)
Externalizing score	5.2 (3.25)	5.0 (3.13)	6.5 (3.03)
Internalizing score	6.3 (3.73)	4.4 (3.15)	8.9 (3.42)
Total Score	11.5 (5.80)	9.4 (5.20)	14.8 (6.21)

[a] 10-item General HRQoL index assessed by the KIDSCREEN-10; [b] Strengths and Difficulties Questionnaire.
